# Oligometastatic versus polymetastatic colon cancer: functional and genomic determinants of divergent metastatic trajectories

**DOI:** 10.37349/etat.2026.1002371

**Published:** 2026-05-13

**Authors:** Roberto Sirica, Carmine Picone, Francesco Sabbatino, Nadia Petrillo, Marco Cascella, Mariachiara Santorsola, Vincenza Granata, Monica Ianniello, Raffaella Ruggiero, Luisa Circelli, Giuliana Ciappina, Massimiliano Berretta, Giovanni Savarese, Alessandro Ottaiano

**Affiliations:** Yong Loo Lin School of Medicine, National University of Singapore, Singapore City, Singapore; ^1^Centro AMES, 80013 Casalnuovo di Napoli, Italy; ^2^Istituto Nazionale Tumori di Napoli, IRCCS “G. Pascale”, 80131 Naples, Italy; ^3^Department of Medicine, Surgery and Dentistry, University of Salerno, 84081 Baronissi, Italy; ^4^Section of Experimental Medicine, Department of Medical Sciences, University of Ferrara, 4412 Ferrara, Italy; ^5^Division of Medical Oncology, “G. Martino” Hospital, 98124 Messina, Italy; ^6^Department of Clinical and Experimental Medicine, University of Messina, 98125 Messina, Italy

**Keywords:** metastatic colon cancer, oligometastatic disease, tumor mutational burden, gene ontology, genotype-phenotype correlation

## Abstract

**Aim:**

The aim of this study is to investigate the molecular and functional features underlying the clinical heterogeneity between oligometastatic (OM) and polymetastatic (PM) colon cancer.

**Methods:**

We performed a genotype-phenotype analysis in a homogeneous cohort of 127 patients with metastatic colon cancer (mCC) profiled using the same next-generation sequencing platform (TruSight Oncology^®^ 500). OM disease was defined as the presence of one to three metastatic lesions per involved organ, involving no more than two organs overall, with all lesions measuring < 70 mm in maximum diameter and no single lesion > 25 mm. Molecular alterations, microsatellite instability (MSI), tumor mutational burden (TMB), and overall survival (OS) were analyzed. Gene Ontology (GO) enrichment and Phenolyzer network analyses were applied to explore functional differences between prognostically distinct molecular subgroups.

**Results:**

OM patients showed a striking survival advantage compared with PM patients [median OS not reached versus 29 months; hazard ratio (HR): 0.20, *P* < 0.0001], validating the clinical distinction between the two phenotypes. PM disease was significantly enriched for *RAS* mutations, whereas OM disease was associated with MSI-high status and elevated TMB. Canonical driver alterations were largely shared between groups, and Phenolyzer analysis revealed similar core oncogenic networks centered on adenomatous polyposis coli (*APC*), tumor protein p53 (*TP53*), and epidermal growth factor receptor (*EGFR*). In contrast, GO analysis demonstrated selective enrichment in PM tumors for molecular functions related to ATP binding, nucleotide binding, and protein kinase activity, consistent with enhanced bioenergetic demand and signaling intensity.

**Conclusions:**

These findings support refined biological stratification of mCC and the exploration of personalized, metastasis-directed strategies, potentially incorporating immunological modulation in OM disease.

## Introduction

Colorectal cancer (CRC) remains one of the major contributors to cancer incidence and mortality in Western countries. Recent global estimates indicate nearly 2 million new CRC diagnoses annually, with close to 1 million deaths worldwide. Incidence rates in high-income regions are among the highest globally, driven by lifestyle factors (e.g., diet, physical inactivity) and modulated by the introduction and expansion of screening programs [1]. Nevertheless, despite preventive strategies and improvements in early detection, CRC-related mortality remains substantial [2].

Metastatic disease accounts for a large proportion of this burden. Approximately 30% of patients present with synchronous metastases, and many others will develop metachronous dissemination during the course of their illness [1, 2]. The liver is the predominant metastatic site, affected in roughly half of all metastatic CRC cases, followed by the lungs, lymph nodes, and peritoneum. The prognosis for stage IV CRC remains poor: although advances in combination chemotherapy, targeted biologics [including anti-vascular endothelial growth factor (VEGF) and anti-epidermal growth factor receptor (EGFR) agents], and metastasis-directed surgical approaches have extended survival for selected patients, a durable cure is uncommon [3].

Historically, metastatic CRC was regarded as a single clinical entity, uniformly characterized by rapid systemic dissemination and poor outcomes. However, accumulating clinical evidence has challenged this uniform view. A subset of patients with limited metastatic burden experience unexpectedly favorable outcomes, including long-term disease control and, in some cases, prolonged survival after local treatment of metastases [4]. In contrast, others exhibit rapid, widespread progression despite intensive systemic therapy. This clinical divergence underpins the concept of oligometastatic (OM) disease, a condition in which the tumor has seeded only a limited number of metastases, often accompanied by more indolent biological behavior that may render curative-intent local interventions feasible [5]. The distinction between OM and polymetastatic (PM) disease has practical and biological implications. Clinically, identifying OM disease can inform decisions regarding surgical resection, stereotactic body radiotherapy (SBRT), or other metastasis-directed strategies. Biologically, the existence of a true OM state points to underlying molecular and microenvironmental factors that constrain metastatic dissemination, as well as to evolutionary and genetic trajectories that favor metastatic containment over widespread progression.

Cancer can be conceptualised as a dynamic evolutionary process, shaped by the interplay between genomic instability, microenvironmental constraints, and therapeutic selective pressures, which collectively drive tumor progression and clinical heterogeneity [6]. Recent advances in genomics and immunology have started to elucidate the molecular architecture of OM CRC. Prior studies suggest that both tumor-intrinsic genetic features and host immunologic characteristics may contribute to this phenotype [7, 8]. In particular, variations in the mutational landscape of primary and metastatic lesions, including regressive trajectories of key driver genes, have been implicated in metastatic containment. Additionally, emerging evidence indicates that the tumor microenvironment and host immune competence may exert selective pressures capable of eliminating or restraining aggressive subclones [8]. Therapy may also modulate the evolutionary dynamics of metastatic disease, with a complex interplay between genomic evolution, treatment-induced selective pressures, and the immune contexture collectively shaping metastatic behavior and, ultimately, the clinical phenotype [9, 10]. However, replication stress—arising from persistent proliferative signaling and defective DNA damage repair—is increasingly recognized as a fundamental driver of tumor evolution and clonal diversification [11]. Excessive stress may promote genomic plasticity and aggressive dissemination, whereas insufficient stress adaptation may activate checkpoint responses and senescence-like programs that constrain expansion. Cellular senescence, together with immune surveillance and microenvironmental pressures, may therefore contribute to maintaining a biologically contained metastatic configuration. Within this framework, OM and PM disease can be interpreted as divergent evolutionary outcomes along axes of genomic stress handling and adaptive resilience, rather than merely quantitative differences in tumor burden.

We explored genotype-phenotype relationships in a well-defined and homogeneous cohort of metastatic colon cancer (mCC) patients. To minimize technical variability, all cases were profiled using the same next-generation sequencing platform. By comparing patients with OM versus PM presentations, our objective was to identify genomic features that may serve as biomarkers of OM behavior and to provide insights with potential relevance for personalized therapeutic approaches.

## Materials and methods

### Patients’ selection and clinical management

This study included patients who underwent genomic profiling at the AMES Center and received treatment consistent with the European Society for Medical Oncology (ESMO) recommendations [12]. All individuals were diagnosed between January 2016 and October 2025. Eligible participants had an Eastern Cooperative Oncology Group (ECOG) Performance Status below 2 and a cachexia risk score under 1 [13]. Patients with peritoneal carcinomatosis or an estimated life expectancy shorter than three months, as judged by their attending physicians, were excluded.

Only adults (≥ 18 years) with mCC were considered. OM disease was defined as mCC characterized by one to three metastatic lesions per involved organ, affecting no more than two organs overall. Eligible lesions were required to measure less than 70 mm in maximum diameter, with no single lesion exceeding 25 mm [4]. To ensure biological consistency of the OM phenotype, patients were confirmed as OM only if they did not develop PM dissemination within one year after the first-line treatment, whether local and/or systemic. Rectal cancer cases were excluded to maintain a uniform study population, given the recognized biological and clinical differences between colon and rectal tumors. In selected cases, following multidisciplinary discussion, local treatments aimed at controlling metastatic sites were performed. These included surgical removal of liver or lung metastases and SBRT to lung lesions, with dose and fractionation tailored to lesion size, location, and surrounding anatomy.

Patients underwent routine clinical surveillance with total-body computed tomography (CT) or magnetic resonance imaging (MRI). Tumor response was categorized using Response Evaluation Criteria in Solid Tumors (RECIST) v1.1 criteria [14]. Disease control included complete response, partial response, or stable disease; progression indicated the absence of disease control.

The study adhered to the ethical standards of the Declaration of Helsinki. At the time of genetic testing, patients provided written consent for future use of anonymized data and biospecimens. Approval was obtained from the AMES Center Institutional Review Board (IRB number CA03/2025).

### Tumor specimens and sequencing

Primary colon cancer samples preserved as formalin-fixed paraffin-embedded (FFPE) blocks were microdissected under morphological guidance. DNA was isolated using the MagCore MGF03 Genomic DNA FFPE One-Step Kit, following the manufacturer’s instructions. DNA quality was assessed in triplicate using the Illumina FFPE QC Kit (San Diego, USA).

Libraries were generated using the TruSight Oncology® 500 (TSO500) panel, which covers 523 cancer-related genes (details in Table S1). This assay detects single nucleotide variants, indels, splice alterations, copy number variations, gene fusions, and immunotherapy biomarkers such as tumor mutational burden (TMB) and microsatellite status. Sequencing was performed on an Illumina NovaSeq 6000.

TMB was calculated according to Chalmers et al. [15], incorporating all coding substitutions and indels—synonymous and nonsynonymous—across 1.9 Mb of target sequence. Variant calling and TMB estimation relied on independent algorithms to enhance reliability (see manufacturer documentation). Microsatellite status was determined using a statistical model based on somatic mutation patterns [16], trained and validated on large The Cancer Genome Atlas (TCGA) cohorts with known microsatellite instability (MSI) status, demonstrating high positive (98.9%) and negative (98.8%) predictive values.

### Bioinformatics analysis and data presentation

Sequencing data were processed using the Illumina TSO500 bioinformatics workflow. The median read count per sample was 117 million, and coverage consistently exceeded the recommended 150× depth. Reads were aligned to the GRCh37 reference genome using the Burrows-Wheeler Aligner [17]. Identified variants were annotated against population and cancer databases, including GENCODE, dbNSFP, International Cancer Genome Consortium-Pan-Cancer Analysis of Whole Genomes (ICGC-PCAWG), Catalogue Of Somatic Mutations In Cancer (COSMIC), 1000Genomes, ClinVar, CancerMine, OncoScore, CIViC, and CBMDB. Variants occurring at a global minor allele frequency < 1% were removed. A four-tier classification system (Tiers 1–4) was applied in accordance with Association for Molecular Pathology (AMP)/American College of Medical Genetics and Genomics (ACMG)/ASCO/CAP consensus recommendations [18, 19], and variants of strong clinical relevance were determined using curated biomarker resources. Overall survival (OS) was used as the study endpoint, defined as the interval between diagnosis of metastatic disease and CRC-related death. Progression-free survival (PFS) was not assessed due to heterogeneous treatment schedules and imaging intervals. Covariates included age (≤ 70 versus > 70 years), sex (male versus female), metastatic burden (one versus multiple sites), response to first-line therapy (disease control versus no control), OM versus PM status, adenomatous polyposis coli (*APC*) mutation status (mutated versus wild-type), *RAS* mutation status (mutated versus wild-type), tumor protein p53 (*TP53*) mutation status (mutated versus wild-type), and TMB category. TMB stratification followed the FDA threshold of > 10 mut/Mb, which has demonstrated predictive value for immunotherapy response [20]. Covariates were selected through consensus discussion with a biostatistician, based on their established clinical-prognostic relevance as well as their distribution and sample size adequacy, to ensure a robust and reliable final model.

Kaplan-Meier curves and log-rank tests were used for univariate OS evaluation. Multivariate analysis employed Cox proportional hazards modelling to assess covariate interactions. Hazard ratios (HRs) and 95% confidence intervals (CIs) were computed, and results were graphically summarized using Forest plots. Continuous variables, such as age, were compared using *t*-tests; categorical variables were assessed with chi-square tests. A *P*-value < 0.05 was considered statistically significant. Analyses were conducted using Microsoft Excel and MedCalc^®^ version 20.112.

### Gene Ontology and Phenolyzer analyses

To explore functional pathways enriched in genes that differed between the two most prognostically distinct groups (OM versus PM), Gene Ontology (GO) enrichment analysis was performed. GO terms were examined across the three standard categories: biological process (BP), molecular function (MF), and cellular component (CC). The analysis was run with the clusterProfiler package (version 4.2.2) in R. A bootstrap approach with 50 iterations was used to mitigate the effect of unequal subgroup sizes. Only GO terms with an adjusted *P*-value < 0.05 were retained.

To further characterize gene interactions, differentially altered genes were analyzed with Phenolyzer, which integrates gene-disease associations, protein networks, signaling pathways, gene families, and regulatory information from databases such as OMIM, Orphanet, ClinVar, GeneReviews, and the GWAS Catalog [21]. A mutation-aware strategy was applied: genes were ranked based on mutation frequency within each subgroup, and the top 20 genes were included in the main analysis. Phenolyzer outputs were used to identify central nodes and network hubs potentially associated with clinical phenotypes. Legends for the generated network diagrams are provided in Figure S1.

## Results

### Clinico-pathological characteristics of OM and PM patient cohorts

Table 1 summarizes the main clinico-pathological characteristics of the analyzed patient cohort, stratified according to metastatic status into OM and PM disease.

**Table 1:** Clinico-pathological characteristics of analysed patients.

**Variable**	**No. (%)**	**OM**	**PM**	***P***
Age	Median: 64 Range: 27–82	Median: 67 Range: 27–81	Median: 62 Range: 31–82	0.0724
Gender				
Female	45 (35.4)	12	33	0.0306
Male	82 (64.6)	38	44	
Side				
Left	82 (64.6)	36	46	0.1598
Right	45 (35.4)	14	31	
Grading				
G1/2	81 (63.8)	40	41	0.0023
G3	46 (36.2)	10	36	
pT				
1/2	14 (11.0)	7	7	0.4268
3	66 (52.0)	35	31	
4	17 (13.4)	6	11	
Unknown	30 (23.6)	2	28	
pN				
0	35 (27.6)	20	15	0.4379
1	38 (29.9)	16	22	
2	24 (18.9)	12	12	
Unknown	30 (23.6)	2	28	
No. of metastatic sites				
1	36 (28.3)	6	30	0.0010
≥ 2	91 (71.7)	44	47	
Liver involvement				
Yes	85 (66.9)	32	53	0.5733
No	42 (33.1)	18	24	
Response to first-line therapy				
Disease control	90 (70.9)	30	60	0.9007
No disease control	25 (19.7)	8	17	
No systemic therapy	12 (9.4)	12	0	
Stereotactic radiotherapy or MWA				
Yes	14 (11.0)	9	5	0.0439
No	113 (89.0)	41	72	
Liver or lung metastasectomy				
Yes	54 (42.5)	35	19	< 0.0001
No	73 (57.5)	15	58	

MWA: microwave ablation; OM: oligometastatic; PM: polymetastatic; pN: pathological nodal status.

The median age of the overall population was 64 years (range: 27–82). Patients in the OM group tended to be older than those in the PM group (median age: 67 versus 62 years, respectively), although this difference did not reach statistical significance (*P* = 0.0724). A significant difference in sex distribution was observed between groups, with a higher proportion of males in the OM cohort compared with the PM cohort (*P* = 0.0306). Primary tumor was predominantly left-sided in the overall population (64.6%), with no statistically significant difference between OM and PM patients (*P* = 0.1598). Tumor grading, however, differed significantly between the two groups: low-to-intermediate grade tumors (G1/2) were more frequent in OM patients, whereas high-grade tumors (G3) were significantly enriched in the PM group (*P* = 0.0023). Pathological T stage distribution did not significantly differ between OM and PM patients (*P* = 0.4268), despite a predominance of pT3 tumors in both groups. Similarly, no significant differences were observed in pathological nodal status (pN) between the two metastatic settings (*P* = 0.4379). The number of metastatic sites differed significantly between the two groups. OM patients were more frequently characterized by a limited number of metastatic sites, whereas PM patients predominantly exhibited involvement of more than two metastatic sites (*P* = 0.0010). Liver involvement was common in both cohorts (66.9% overall) and did not differ significantly between OM and PM patients (*P* = 0.5733). Response to first-line systemic therapy was comparable between the two groups, with disease control achieved in approximately 71% of treated patients and no significant difference observed (*P* = 0.9007). Notably, a subset of OM patients did not receive systemic therapy. Locoregional treatment approaches differed between the two metastatic phenotypes. The use of SBRT or microwave ablation (MWA) was significantly more frequent in OM patients compared with PM patients (*P* = 0.0439). Moreover, surgical resection of liver or lung metastases was markedly more common in the OM cohort, whereas PM patients were significantly less likely to undergo metastasectomy (*P* < 0.0001).

### Clinical outcome validation and molecular stratification of OM versus PM disease

**Figure 1:**
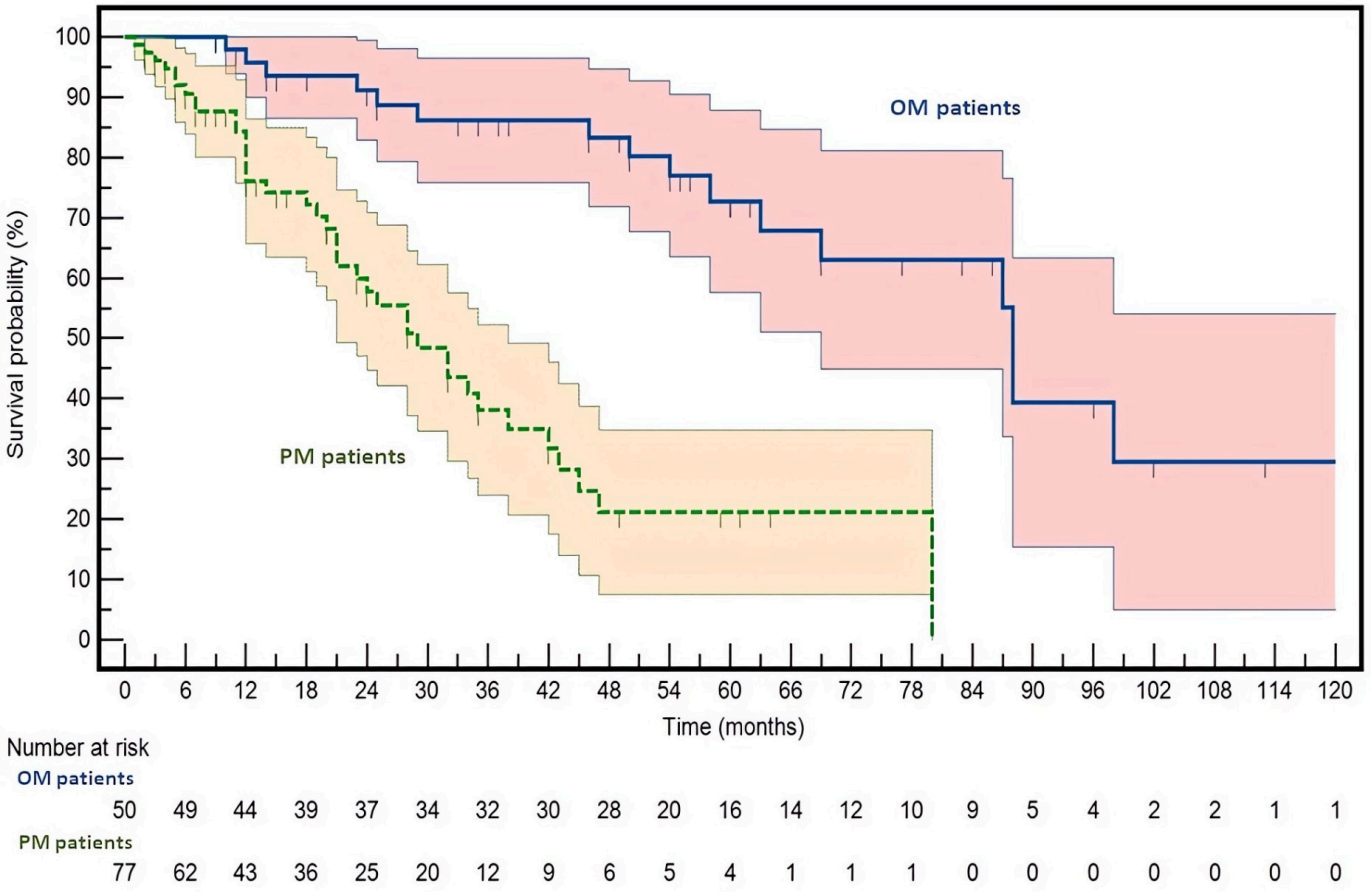
**Kaplan-Meier survival curves for oligometastatic (OM) and polymetastatic (PM) colorectal cancer patients.** Survival probability (Y-axis) is plotted over time (X-axis) with 95% confidence intervals (CIs). OM patients are shown in dark blue, and PM patients in green. Hazard ratio (HR) and 95% CI are reported in the results section. The survival curves are significantly different, with a log-rank *P* < 0.0001.

Consistent with the expected clinical behavior of OM, this cohort exhibited a markedly superior OS compared with the PM cohort, thereby supporting the appropriateness and robustness of the patients’ selection (Figure 1).

After a median follow-up of 52 months, a total of 55 death events were recorded (OM versus PM: 16/50 versus 39/77). Median OS was not reached in the OM group, whereas it was 29 months in PM patients. Survival analysis confirmed a highly significant difference between the two cohorts, with OM patients experiencing a substantially reduced risk of death (HR = 0.1974; 95% CI: 0.1095–0.3559; *P* < 0.0001 by log-rank test). This pronounced survival advantage reinforces the notion that long-term survival represents a defining clinical hallmark of the OM phenotype and confirms that the two populations are well differentiated from a prognostic standpoint. A multivariate analysis confirmed that the prognostic impact of the OM phenotype is strong and remains independent of other clinical and/or molecular variables (Table S2).

Building upon this validated clinical stratification, we next investigated whether such phenotypic divergence was mirrored by differences in the distribution of key molecular biomarkers implicated as oncogenic drivers in this disease (Table 2).

**Table 2:** Distribution of key molecular alterations stratified by metastatic status.

**Molecular variable**	**No. (%)**	**OM**	**PM**	***P***
*APC* status				
Wt	40 (31.5)	10	30	0.0250
Mut	87 (68.5)	40	47	
*BRAF* p.V600E status				
Absent	118 (92.9)	49	69	0.0730
Present	9 (7.1)	1	8	
*BRAF* all mutations				
Wt	115 (90.6)	47	68	0.2862
Mut	12 (9.4)	3	9	
*ERBB2* amplification				
Yes	10 (7.9)	2	8	0.1932
No	117 (92.1)	48	69	
*PIK3CA* status				
Wt	92 (72.4)	35	57	0.6212
Mut	35 (27.6)	15	20	
*RAS* status				
Wt	69 (54.3)	33	36	0.0341
Mut	58 (45.7)	17	41	
*TP53* status				
Wt	43 (33.9)	14	29	0.2628
Mut	84 (66.1)	36	48	
MSS				
Stable	117 (92.1)	42	75	0.0064
Unstable	10 (7.9)	8	2	
TMB (mut/Mb)				
≥ 10	35 (27.6)	19	16	0.0345
< 10	92 (72.4)	31	61	

*APC*: adenomatous polyposis coli; *BRAF*: v-Raf murine sarcoma viral oncogene homolog B1; MSS: microsatellite stability; *PIK3CA*: phosphatidylinositol-4,5-bisphosphate 3-kinase catalytic subunit alpha; *RAS*: Rat sarcoma viral oncogene family (including KRAS and NRAS); TMB: tumor mutational burden; *TP53*: tumor protein p53; OM: oligometastatic; PM: polymetastatic; *ERBB2*: v-Erb-B2 Avian Erythroblastic Leukemia Viral Oncogene Homolog 2; Wt: wild-type.

While most molecular alterations were similarly distributed between OM and PM patients, several noteworthy patterns emerged. v-Raf murine sarcoma viral oncogene homolog B1 (*BRAF*) p.V600E mutations were numerically more frequent in the PM cohort (8 versus 1 cases), although this difference did not reach statistical significance (*P* = 0.0730). Likewise, v-Erb-B2 Avian Erythroblastic Leukemia Viral Oncogene Homolog 2 (*ERBB2*) amplification, phosphatidylinositol-4,5-bisphosphate 3-kinase catalytic subunit alpha (*PIK3CA*) mutations, and *TP53* alterations showed no significant differences between groups. In contrast, *APC* mutations were significantly more prevalent in OM disease, whereas *APC* wild-type tumors were relatively enriched in the PM group (*P* = 0.0250). *RAS* mutations, conversely, were significantly more frequent in PM patients compared with OM patients (41 versus 17 cases; *P* = 0.0341). Microsatellite stability (MSS) status also differed markedly between cohorts, with MSI-high tumors being significantly enriched in OM patients (*P* = 0.0064). Consistently, a high TMB (TMB ≥ 10 mut/Mb) was more commonly observed in the OM group, whereas low TMB (< 10 mut/Mb) predominated in PM patients (*P* = 0.0345).

### Genotype-phenotype correlation with Phenolyzer and GO analyses

To further characterize the mutational landscape associated with metastatic burden, we analyzed the 20 genes harboring the most frequent Tiers 1–3 mutations in the two study groups. Within this framework, we specifically focused on identifying group-restricted alterations, herein referred to as private mutational events, defined as mutations exclusively detected in one group but absent in the other. Using this approach, exclusive mutations in *ERCC1*, *MST1*, myeloblastosis oncogene (*MYB*), and nuclear receptor corepressor 1 (*NCOR1*) were identified in the PM cohort, whereas *FAT1*, isocitrate dehydrogenase 2 (*IDH2*), low-density lipoprotein receptor-related protein 1B (*LRP1B*), and paired box 8 (*PAX8*) emerged as exclusively mutated genes in the OM group. The gene-specific mutational burden for these private events across the analyzed cohort is reported in Table S3 and Table S4.

**Figure 2:**
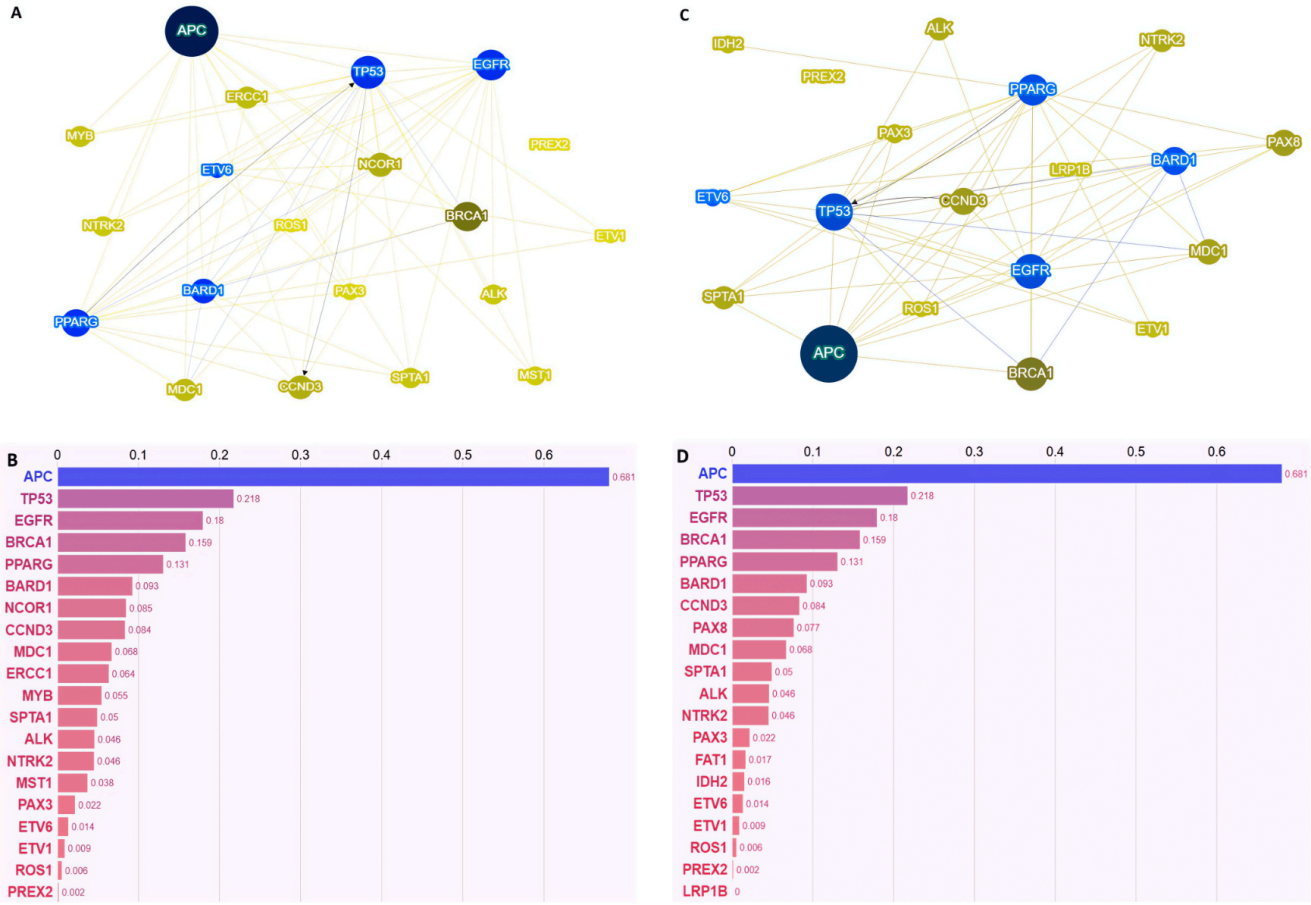
**Phenolyzer-based molecular characterization of polymetastatic (PM) and oligometastatic (OM) colorectal cancer.** Panels A and C show the interaction networks generated by Phenolyzer for PM (**A**) and OM (**C**) patients, highlighting the functional interconnections among prioritized genes in each metastatic phenotype. Nodes represent genes, and edges indicate predicted functional or regulatory interactions. Panels B and D display the corresponding gene prioritization as bar plots for PM (**B**) and OM (**D**) patients. In both panels, the Y-axis lists the genes, and the X-axis shows the corresponding Phenolyzer scores. The bars represent the Phenolyzer score for each gene, reflecting both its predicted relevance to metastatic colorectal cancer and its connectivity within the interaction network (see methods).

To explore potential functional or biological relationships among the genes most frequently altered in the two groups, a Phenolyzer-based network analysis was performed (Figure 2).

Despite the qualitative differences in the genes identified as group-specific, this analysis did not reveal distinct relational or network patterns between the two metastatic settings. In both cohorts, *APC*, *TP53*, and *EGFR* consistently emerged as the most highly interconnected nodes within the gene interaction network, indicating a shared core of molecular interdependencies across different metastatic phenotypes.

**Figure 3:**
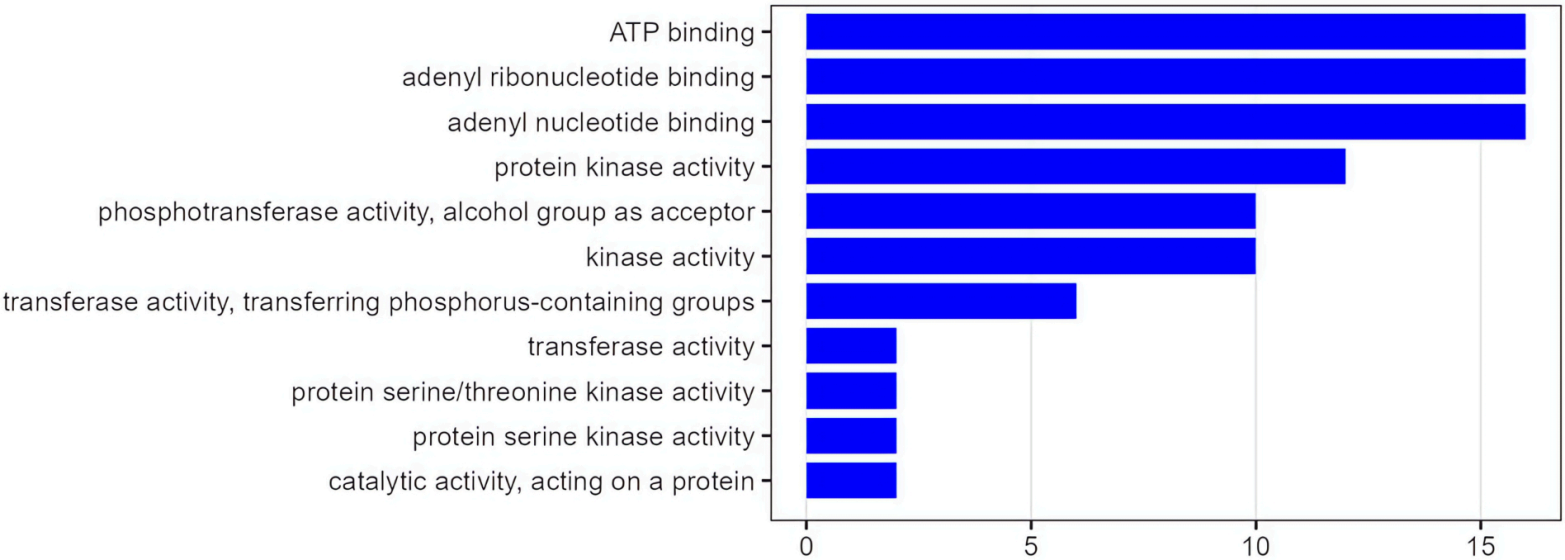
**Gene Ontology enrichment analysis comparing poly-metastatic and oligometastatic colorectal cancer.** The figure shows statistically significant enriched pathways (Y-axis) plotted against the enrichment score (X-axis), which quantifies the degree of pathway overrepresentation in poly-metastatic compared with oligo-metastatic disease.

GO analysis revealed a selective enrichment of molecular characteristics related to cellular energy handling and signal transduction in the PM versus OM patients (Figure 3). Specifically, the most significantly overrepresented GO terms belonged to the MF category and included ATP binding, adenyl ribonucleotide binding, adenyl nucleotide binding, and protein kinase activity.

## Discussion

The concept of OM disease has progressively evolved from a purely descriptive clinical entity into a biologically grounded state, characterized by limited metastatic dissemination and a more indolent natural history compared with widespread PM disease [10]. In this study, we sought to interrogate the clinical, molecular, and functional correlates of OM versus PM mCC in order to better understand whether these two phenotypes represent quantitatively different stages along a metastatic continuum or qualitatively distinct biological conditions. Our results provide several important suggestions that collectively support the latter hypothesis, while simultaneously highlighting the complexity underlying metastatic behavior. However, while our data are consistent with a biologically distinct OM configuration, they should not be interpreted as demonstrating biological inevitability; rather, they suggest probabilistic tendencies within a dynamic evolutionary landscape.

From a clinical standpoint, the marked survival advantage observed in OM patients represents a critical internal validation of cohort selection. The median OS was not reached in the OM group, in contrast to a median survival of 29 months in PM patients, with a nearly 80% relative reduction in the risk of death. This pronounced difference is consistent with the growing body of literature indicating that long-term survival is a defining hallmark of OM disease and reinforces the notion that OM is not merely an early temporal snapshot of PM progression [5]. Rather, the observed outcomes strongly suggest that OM patients harbor intrinsic disease features that limit metastatic competence and/or enable prolonged disease control through local and systemic interventions. At the same time, it remains plausible that OM disease represents a metastable equilibrium—an intermediate evolutionary state maintained by a balance between tumor expansion and host-imposed constraints—rather than a permanently fixed biological endpoint.

Clinico-pathological characteristics further support this interpretation. OM tumors were significantly enriched for low-to-intermediate histological grade, whereas high-grade tumors were overrepresented in the PM cohort, consistent with a more aggressive biological phenotype. Importantly, no significant differences were observed in primary tumor T stage or nodal involvement, underscoring that the divergent metastatic behavior cannot be simply attributed to differences in local tumor extent at diagnosis. Instead, metastatic burden appears to reflect distinct systemic disease properties that manifest downstream of primary tumor growth. The higher utilization of locoregional approaches, such as metastasectomy, stereotactic radiotherapy, and ablative techniques, in OM patients is likely a consequence, rather than a cause, of this more favorable biology, enabling durable disease control when metastatic dissemination remains limited.

Against this clinically validated backdrop, we examined whether molecular differences might underlie the divergent metastatic phenotypes. At the level of canonical driver alterations, several noteworthy patterns emerged. *APC* mutations were significantly enriched in OM disease, whereas *RAS* mutations were significantly more frequent in PM disease; *BRAF* p.V600E mutations also showed a numerical enrichment in PM patients, in line with their well-established association with aggressive tumor biology and poor prognosis [22]. However, aside from these alterations, most established driver events—including *ERBB2* amplification, *PIK3CA* mutations, and *TP53* alterations—were comparably distributed between OM and PM cohorts. These findings suggest that while certain oncogenic drivers may contribute to metastatic aggressiveness, driver dominance alone is insufficient to fully explain the stark clinical divergence between the two groups.

More revealing was the differential distribution of MSS and TMB. MSI-high status and elevated TMB were significantly enriched in the OM cohort, whereas PM disease was predominantly characterized by MSS and low mutational burden. This observation is particularly intriguing, as it shifts the interpretative focus away from individual oncogenic lesions toward broader genomic and immunological landscapes. MSI-high tumors are known to generate a higher neoantigen load, leading to enhanced immune recognition and, in many cases, more effective immune-mediated tumor control [23, 24]. In this context, the association between OM disease, MSI, and high TMB raises the possibility that immune surveillance may play a central role in constraining metastatic dissemination, thereby maintaining an OM state. However, this immune-surveillance hypothesis has not been directly tested in tumor tissues from this cohort and should therefore be considered inferential. Dedicated studies and prospective validation are required to substantiate this interpretation. Nevertheless, immune editing is inherently dynamic, and immune-mediated containment may fluctuate over time, potentially allowing eventual escape and transition toward more aggressive phenotypes. Within this evolving framework, additional components of the tumor microenvironment may further modulate the balance between containment and dissemination. Tumor-associated macrophages, for instance, represent key regulators of immune contexture, and their polarization toward an M2-like phenotype has been implicated in immune suppression and tumor progression, including in CRC [25]. In parallel, metastatic dissemination is increasingly recognized as a process sustained by complex intercellular communication networks, including extracellular vesicle-mediated signaling between tumor cells and the surrounding microenvironment. These vesicles can influence pre-metastatic niche formation, immune modulation, and organotropism, thereby contributing to metastatic competence [26]. Integrating these perspectives may help refine the biological interpretation of OM versus PM states, extending beyond intrinsic tumor features to encompass dynamic microenvironmental and systemic interactions.

Despite identifying group-specific “private” mutational events, such as *ERCC1*, *MST1*, *MYB*, and *NCOR1* in PM disease and *FAT1*, *IDH2*, *LRP1B*, and PAX8 in OM disease, network-based analysis using Phenolyzer revealed no meaningful differences in the relational architecture of altered genes between the two cohorts. However, the Phenolyzer-based network analysis should be interpreted as exploratory and hypothesis-generating rather than mechanistically definitive. While it enables a systems-level prioritization of genes and functional interconnections associated with distinct metastatic phenotypes, it does not establish causal biological relationships. Dedicated in vitro and in vivo investigations will be required to validate the functional relevance of the identified candidate genes and enriched pathways, and to clarify their potential contribution to metastatic competence and disease trajectory. Among the private genes identified, *ERCC1* emerges as the only alteration directly involved in the maintenance of genomic integrity, given its central role in nucleotide excision repair and interstrand crosslink repair. Dysfunction of *ERCC1* is mechanistically linked to defective DNA damage resolution and the accumulation of genomic lesions, thereby promoting genomic instability. However, it is important to note that *ERCC1* loss may be at least partially functionally compensated by alternative DNA repair pathways or redundant repair complexes, potentially attenuating its phenotypic impact on tumor behavior in certain molecular contexts [24, 27]. In contrast, *LRP1B* does not act as a primary driver of genomic instability but rather represents a marker of an intrinsically unstable genomic background. *LRP1B*-mutant tumors are noteworthy for exhibiting a higher TMB and neoantigen load, along with upregulation of antigen-presentation and inflammatory gene signatures and increased CD4⁺/CD8⁺ T-cell infiltration—features that plausibly enhance tumor immunogenicity and have been associated with improved clinical courses [28–30]. Despite these differences, in both OM and PM disease, *APC*, *TP53*, and *EGFR* emerged as the most highly interconnected nodes, reflecting a shared core of molecular dependencies typical of CRC biology. This convergence suggests that, at a systems level, both phenotypes rely on similar oncogenic frameworks, despite diverging clinically. Importantly, in the absence of mechanistic validation, the identification of these biological drivers should be interpreted as hypothesis-generating rather than definitive.

Furthermore, GO enrichment analysis revealed a selective functional skewing in PM disease toward molecular programs related to energy utilization and signal transduction. The overrepresentation of GO terms such as ATP binding, adenyl ribonucleotide/nucleotide binding, and protein kinase activity points to an increased reliance on energy-dependent processes and kinase-driven signaling cascades in PM tumors. Collectively, these features are hallmarks of heightened proliferative capacity, metabolic adaptability, and dynamic signal integration, all of which are critical for sustaining widespread metastatic growth across multiple anatomical sites. Interestingly, PM tumors display a kinase-enriched, RAS-skewed, and bioenergetically demanding molecular profile that is permissive for sustained Nuclear Factor kappa-light-chain-enhancer of activated B cells (NF-κB) activation, potentially reinforcing survival signaling, stress tolerance, metastatic plasticity, and stemness [31]. However, it should be emphasized that this inference is based on functional enrichment rather than direct measurement of pathway activation, and alternative explanations—such as microenvironmental cytokine gradients, stromal interactions, or hypoxia-induced signaling—may also contribute to this phenotype. In contrast, the absence of comparable GO enrichment signals in OM disease aligns with a less metabolically and signaling-intensive phenotype. This relative functional restraint is coherent with the clinical and genomic features observed in OM patients, including higher MSI/TMB and prolonged survival, and may reflect a tumor state that is more effectively constrained by immune surveillance and less dependent on aggressive bioenergetic and kinase-driven programs. Yet, this restrained profile may not represent a stable biological endpoint; rather, it could correspond to a transient balance between proliferative drive and external constraint, susceptible to disruption by additional genomic events or environmental pressures. Importantly, these GO-level differences emerge despite a shared core oncogenic network, suggesting that metastatic behavior may be modulated not by distinct pathway architectures, but by differential functional weighting of common molecular components. Such modulation likely reflects an interplay between intrinsic tumor cell programs and extrinsic forces, including immune editing, stromal conditioning, vascular dynamics, and therapy-induced stress. Overall, the GO analysis provides a functional bridge between genomic observations and clinical behavior, indicating that PM disease could be characterized by a molecular bias toward energy-intensive and kinase-mediated processes that plausibly support extensive metastatic competence. Conversely, OM disease appears to lack this functional enrichment, reinforcing the notion that OM biology could represent a qualitatively distinct, system-level state rather than a mere quantitative reduction of metastatic spread.

Collectively, these observations may argue against an overly simplistic model in which OM disease is driven by a unique set of dominant oncogenic pathways. Rather, they could support a more nuanced interpretation in which OM and PM disease diverge through higher-order biological mechanisms integrating genomic complexity, tumor-host interactions, and immune dynamics. Within this framework, driver mutations may contribute to establishing the oncogenic potential of the tumor, while the ultimate metastatic trajectory might be influenced by additional layers of regulation that extend beyond the cancer cell itself.

Several limitations of this study should be acknowledged. The retrospective nature of the analysis and the sample size may limit the statistical power to detect subtle molecular differences, particularly for low-frequency events. Moreover, our analyses were based primarily on genomic data, without direct assessment of immune infiltration, spatial heterogeneity, or functional immune activity. An additional limitation of the genotype-phenotype analysis is the absence of validation in an independent or publicly available dataset; therefore, external validation in future studies is warranted. Another point deserving consideration concerns the interpretation of bioinformatic enrichment analyses derived from large transcriptomic repositories and curated genomic datasets. Differential expression and pathway enrichment results may be influenced by technical and compositional biases inherent to bulk data, including sample heterogeneity and variability in preprocessing pipelines. These factors can introduce distortions that affect biological inference and limit the robustness of conclusions. Accordingly, our findings should be interpreted with caution, acknowledging the intrinsic limitations of bulk transcriptomic analyses as highlighted in prior methodological studies [32, 33]. Future prospective studies integrating multi-omics approaches—including transcriptomic, proteomic, immunophenotypic, and spatial profiling analyses—will be essential to more comprehensively define the biological basis of the OM phenotype. Applied to well-annotated and adequately powered cohorts, such integrative strategies would enable a more in-depth functional characterization of the candidate genes and molecular networks emerging from our findings, thereby clarifying their biological significance and potential clinical implications. Importantly, although our analysis captures baseline genomic features of primary tumors, metastatic behavior likely emerges from a dynamic interplay between intrinsic genomic architecture and subsequent stress-adaptive responses induced by treatment and other external exposures. A more comprehensive characterization of these external interferences, including systemic therapies, locoregional interventions, inflammatory cues, metabolic stress, and even non-oncologic perioperative factors, will, therefore, be necessary. Such influences may reshape tumor cell states, modulate stem-like compartments, and alter stress tolerance and survival programs, ultimately impacting metastatic trajectories [34]. Disentangling baseline genomic determinants from environmentally induced adaptive reprogramming will be critical to achieve a biologically integrated understanding of OM versus PM evolution.

In conclusion, our findings reinforce the concept that OM and PM CRC represent clinically and biologically distinct entities. While differences in specific driver alterations are detectable, they do not fully account for the profound divergence in clinical outcomes. Instead, broader biological and immunological features, closely linked to MSI status and TMB, appear to play a pivotal role in shaping metastatic behavior. Advancing our understanding of these mechanisms will be critical not only for refining prognostic stratification but also for identifying patients in whom limited metastatic disease truly reflects an indolent, controllable state rather than an early manifestation of aggressive systemic spread.

## Abbreviations

ACMG: American College of Medical Genetics and Genomics
AMP: Association for Molecular Pathology
APC: adenomatous polyposis coli
BP: biological process
BRAF: v-Raf murine sarcoma viral oncogene homolog B1
CC: cellular component
CIs: confidence intervals
COSMIC: Catalogue Of Somatic Mutations In Cancer
CRC: colorectal cancer
CT: computed tomography
ECOG: Eastern Cooperative Oncology Group
EGFR: epidermal growth factor receptor
ERBB2: v-Erb-B2 Avian Erythroblastic Leukemia Viral Oncogene Homolog 2
FFPE: formalin-fixed paraffin-embedded
GO: Gene Ontology
HR: hazard ratio
ICGC-PCAWG: International Cancer Genome Consortium-Pan-Cancer Analysis of Whole Genomes
IDH2: isocitrate dehydrogenase 2
IRB: Institutional Review Board
LRP1B: low-density lipoprotein receptor-related protein 1B
mCC: metastatic colon cancer
MF: molecular function
MSI: microsatellite instability
MSS: microsatellite stability
MWA: microwave ablation
MYB: myeloblastosis oncogene
NCOR1: nuclear receptor corepressor 1
NF-κB: Nuclear Factor kappa-light-chain-enhancer of activated B cells
OM: oligometastatic
OS: overall survival
PAX8: paired box 8
PIK3CA: phosphatidylinositol-4,5-bisphosphate 3-kinase catalytic subunit alpha
PM: polymetastatic
RECIST: Response Evaluation Criteria in Solid Tumors
SBRT: stereotactic body radiotherapy
TCGA: The Cancer Genome Atlas
TMB: tumor mutational burden
TP53: tumor protein p53
VEGF: vascular endothelial growth factor

## Supplementary materials

The supplementary materials for this article are available at: https://www.explorationpub.com/uploads/Article/file/1002371_sup_1.pdf.

## Declarations

### Availability of data and materials

Raw sequencing data have been deposited in the European Nucleotide Archive (ENA) under the accession numbers reported at https://zenodo.org/records/18845198 (last accessed March 3, 2026).

### Copyright

© The Author(s) 2026.

## References

[1] Sung H, Ferlay J, Siegel RL, Laversanne M, Soerjomataram I, Jemal A (2021). Global Cancer Statistics 2020: GLOBOCAN Estimates of Incidence and Mortality Worldwide for 36 Cancers in 185 Countries. CA Cancer J Clin.

[2] Capuozzo M, Picone C, Sabbatino F, Santorsola M, Caraglia F, Iervolino D (2025). Genetic, Epidemiological, Clinical, and Therapeutic Trajectories in Colon and Rectal Cancers. Cancers (Basel).

[3] Cervantes A, Adam R, Roselló S, Arnold D, Normanno N, Taïeb J, ESMO Guidelines Committee (2023). Metastatic colorectal cancer: ESMO Clinical Practice Guideline for diagnosis, treatment and follow-up. Ann Oncol.

[4] Ottaiano A, Santorsola M, Circelli L, Trotta AM, Izzo F, Perri F (2023). Oligo-Metastatic Cancers: Putative Biomarkers, Emerging Challenges and New Perspectives. Cancers (Basel).

[5] Lievens Y, Guckenberger M, Gomez D, Hoyer M, Iyengar P, Kindts I (2020). Defining oligometastatic disease from a radiation oncology perspective: An ESTRO-ASTRO consensus document. Radiother Oncol.

[6] Sonkin D, Thomas A, Teicher BA (2024). Cancer treatments: Past, present, and future. Cancer Genet.

[7] Ottaiano A, Nasti G, Santorsola M, Altieri V, Di Fruscio G, Circelli L (2021). KRAS Mutational Regression Is Associated With Oligo-Metastatic Status and Good Prognosis in Metastatic Colorectal Cancer. Front Oncol.

[8] Ottaiano A, de Vera d’Aragona RP, Trotta AM, Santorsola M, Napolitano M, Scognamiglio G (2022). Characterization of KRAS Mutational Regression in Oligometastatic Patients. Front Immunol.

[9] Gatenby RA, Brown JS (2020). Integrating evolutionary dynamics into cancer therapy. Nat Rev Clin Oncol.

[10] Weichselbaum RR, Hellman S (2011). Oligometastases revisited. Nat Rev Clin Oncol.

[11] Ray SK, Mukherjee S (2025). Exploring replication stress and cellular senescence as key targets in novel cancer therapies. Cancer Genet.

[12] Van Cutsem E, Cervantes A, Adam R, Sobrero A, Van Krieken JH, Aderka D (2016). ESMO consensus guidelines for the management of patients with metastatic colorectal cancer. Ann Oncol.

[13] Martin L, Senesse P, Gioulbasanis I, Antoun S, Bozzetti F, Deans C (2015). Diagnostic criteria for the classification of cancer-associated weight loss. J Clin Oncol.

[14] Eisenhauer EA, Therasse P, Bogaerts J, Schwartz LH, Sargent D, Ford R (2009). New response evaluation criteria in solid tumours: revised RECIST guideline (version 1.1). Eur J Cancer.

[15] Chalmers ZR, Connelly CF, Fabrizio D, Gay L, Ali SM, Ennis R (2017). Analysis of 100,000 human cancer genomes reveals the landscape of tumor mutational burden. Genome Med.

[16] Cortes-Ciriano I, Lee S, Park WY, Kim TM, Park PJ (2017). A molecular portrait of microsatellite instability across multiple cancers. Nat Commun.

[17] Li H, Durbin R (2009). Fast and accurate short read alignment with Burrows-Wheeler transform. Bioinformatics.

[18] Li MM, Datto M, Duncavage EJ, Kulkarni S, Lindeman NI, Roy S (2017). Standards and Guidelines for the Interpretation and Reporting of Sequence Variants in Cancer: A Joint Consensus Recommendation of the Association for Molecular Pathology, American Society of Clinical Oncology, and College of American Pathologists. J Mol Diagn.

[19] Li MM, Cottrell CE, Pullambhatla M, Roy S, Temple-Smolkin RL, Turner SA (2023). Assessments of Somatic Variant Classification Using the Association for Molecular Pathology/American Society of Clinical Oncology/College of American Pathologists Guidelines: A Report from the Association for Molecular Pathology. J Mol Diagn.

[20] Palmeri M, Mehnert J, Silk AW, Jabbour SK, Ganesan S, Popli P (2022). Real-world application of tumor mutational burden-high (TMB-high) and microsatellite instability (MSI) confirms their utility as immunotherapy biomarkers. ESMO Open.

[21] Yang H, Robinson PN, Wang K (2015). Phenolyzer: phenotype-based prioritization of candidate genes for human diseases. Nat Methods.

[22] Xu T, Li J, Wang Z, Zhang X, Zhou J, Lu Z (2023). Real-world treatment and outcomes of patients with metastatic BRAF mutant colorectal cancer. Cancer Med.

[23] Wilbur HC, Le DT, Agarwal P (2024). Immunotherapy of MSI Cancer: Facts and Hopes. Clin Cancer Res.

[24] Wang Q, Yu M, Zhang S (2025). The characteristics of the tumor immune microenvironment in colorectal cancer with different MSI status and current therapeutic strategies. Front Immunol.

[25] Zhang J, Xu Y, Han X, Gao Y, Wei Z, Sun X (2025). Galectin-9 promotes colon cancer development by polarizing macrophages toward the M2 phenotype. Cancer Genet.

[26] Jahangiri L (2025). The impact of extracellular vesicles on breast cancer metastasis and therapeutics: genetic considerations. Cancer Genet.

[27] Helleday T, Petermann E, Lundin C, Hodgson B, Sharma RA (2008). DNA repair pathways as targets for cancer therapy. Nat Rev Cancer.

[28] de Almeida LC, Calil FA, Machado-Neto JA, Costa-Lotufo LV (2021). DNA damaging agents and DNA repair: From carcinogenesis to cancer therapy. Cancer Genet.

[29] Chen H, Chong W, Wu Q, Yao Y, Mao M, Wang X (2019). Association of *LRP1B* Mutation With Tumor Mutation Burden and Outcomes in Melanoma and Non-small Cell Lung Cancer Patients Treated With Immune Check-Point Blockades. Front Immunol.

[30] He Z, Feng W, Wang Y, Shi L, Gong Y, Shi Y (2023). LRP1B mutation is associated with tumor immune microenvironment and progression-free survival in lung adenocarcinoma treated with immune checkpoint inhibitors. Transl Lung Cancer Res.

[31] Tan S, Chen Y, Chen Y, Liu S, Yang C, Mi Y (2026). HOXC8-activated TRIM22/NF-κB pathway promotes stemness in colorectal cancer. Cancer Lett.

[32] Liu H, Guo Z, Wang P (2024). Genetic expression in cancer research: Challenges and complexity. Gene Rep.

[33] Liu H, Li Y, Karsidag M, Tu T, Wang P (2025). Technical and Biological Biases in Bulk Transcriptomic Data Mining for Cancer Research. J Cancer.

[34] Liu H (2020). A prospective for the potential effect of local anesthetics on stem-like cells in colon cancer. Biomed J Sci Tech Res.

