# Supplementary material for: Oligometastatic versus polymetastatic colon cancer: functional and genomic determinants of divergent metastatic trajectories
Source: Explor Target Antitumor Ther. 2026 May 13;7:1002371. doi: 10.37349/etat.2026.1002371 (PMC13176761; doi:10.37349/etat.2026.1002371)
Supplement: Supplementary file 1 [file 1002371_sup_1.pdf]

Table S1. List of studied genes.

**Genes**
ABL1, ABL2, ACVR1, ACVR1B, AKT1, AKT2, AKT3, ALK, ALOX12B, ANKRD11, ANKRD26, APC, AR, ARAF, ARFRP1, ARID1A,
ARID1B, ARID2, ARID5B, ASXL1, ASXL2, ATM, ATR, ATRX, AURKA, AURKB, AXIN1, AXIN2, AXL, B2M, BAP1, BARD1, BBC3,
BCL10, BCL2, BCL2L1, BCL2L11, BCL2L2, BCL6, BCOR, BCORL1, BCR, BIRC3, BLM, BMPR1A, BRAF, BRCA1, BRCA2, BRD4,
BRIP1, BTG1, BTK, C11orf30, CALR, CARD11, CASP8, CBFB, CBL, CCND1, CCND2, CCND3, CCNE1, CD274, CD276, CD74,
CD79A, CD79B, CDC73, CDH1, CDK12, CDK4, CDK6, CDK8, CDKN1A, CDKN1B, CDKN2A, CDKN2B, CDKN2C, CEBPA, CENPA,
CHD2, CHD4, CHEK1, CHEK2, CIC, CREBBP, CRKL, CRLF2, CSF1R, CSF3R, CSNK1A1, CTCF, CTLA4, CTNNA1, CTNNB1, CUL3,
CUX1, CXCR4, CYLD, DAXX, DCUN1D1, DDR2, DDX41, DHX15, DICER1, DIS3, DNAJB1, DNMT1, DNMT3A, DNMT3B, DOT1L,
E2F3, EED, EGFL7, EGFR, EIF1AX, EIF4A2, EIF4E, EML4, EP300, EPCAM, EPHA3, EPHA5, EPHA7, EPHB1, ERBB2, ERBB3,
ERBB4, ERCC1, ERCC2, ERCC3, ERCC4, ERCC5, ERG, ERRFI1, ESR1, ETS1, ETV1, ETV4, ETV5, ETV6, EWSR1, EZH2, FAM123B,
FAM175A, FAM46C, FANCA, FANCC, FANCD2, FANCE, FANCF, FANCG, FANCI, FANCL, FAS, FAT1, FBXW7, FGF1, FGF10,
FGF14, FGF19, FGF2, FGF23, FGF3, FGF4, FGF5, FGF6, FGF7, FGF8, FGF9, FGFR1, FGFR2, FGFR3, FGFR4, FH, FLCN, FLI1,
FLT1, FLT3, FLT4, FOXA1, FOXL2, FOXO1, FOXP1, FRS2, FUBP1, FYN, GABRA6, GATA1, GATA2, GATA3, GATA4, GATA6, GID4,
GLI1, GNA11, GNA13, GNAQ, GNAS, GPR124, GPS2, GREM1, GRIN2A, GRM3, GSK3B, H3F3A, H3F3B, H3F3C, HGF, HIST1H1C,
HIST1H2BD, HIST1H3A, HIST1H3B, HIST1H3C, HIST1H3E, HIST1H3F, HIST1H3G, HIST1H3H, HIST1H3I, HIST1H3J, HIST2H3A,
HIST2H3C, HIST2H3D, HIST3H3, HLA-A, HLA-B, HLA-C, HNF1A, HNRNPK, HOXB13, HRAS, HSD3B1, HSP90AA1, ICOSLG, ID3,
IDH1, IDH2, IFNGR1, IGF1, IGF1R, IGF2, IKBKE, IKZF1, IL10, IL7R, INHA, INHBA, INPP4A, INPP4B, INSR, IRF2, IRF4, IRS1,
IRS2, JAK1, JAK2, JAK3, JUN, KAT6A, KDM5A, KDM5C, KDM6A, KDR, KEAP1, KEL, KIF5B, KIT, KLF4, KLHL6, KMT2B, KMT2C,
KMT2D, KRAS, LAMP1, LATS1, LATS2, LMO1, LRP1B, LYN, LZTR1, MAGI2, MALT1, MAP2K1, MAP2K2, MAP2K4, MAP3K1,
MAP3K13, MAP3K14, MAP3K4, MAPK1, MAPK3, MAX, MCL1, MDC1, MDM2, MDM4, MED12, MEF2B, MEN1, MET, MGA, MITF,
MLH1, MLL, MLLT3, MPL, MRE11A, MSH2, MSH3, MSH6, MST1, MST1R, MTOR, MUTYH, MYB, MYC, MYCL1, MYCN, MYD88,

MYOD1, NAB2, NBN, NCOA3, NCOR1, NEGR1, NF1, NF2, NFE2L2, NFKBIA, NKX2-1, NKX3-1, NOTCH1, NOTCH2, NOTCH3, NOTCH4, NPM1, NRAS, NRG1, NSD1, NTRK1, NTRK2, NTRK3, NUP93, NUTM1, PAK1, PAK3, PAK7, PALB2, PARK2, PARP1, PAX3, PAX5, PAX7, PAX8, PBRM1, PDCD1, PDCD1LG2, PDGFRA, PDGFRB, PDK1, PDPK1, PGR, PHF6, PHOX2B, PIK3C2B, PIK3C2G, PIK3C3, PIK3CA, PIK3CB, PIK3CD, PIK3CG, PIK3R1, PIK3R2, PIK3R3, PIM1, PLCG2, PLK2, PMAIP1, PMS1, PMS2, PNRC1, POLD1, POLE, PPARG, PPM1D, PPP2R1A, PPP2R2A, PPP6C, PRDM1, PREX2, PRKAR1A, PRKCI, PRKDC, PRSS8, PTCH1, PTEN, PTPN11, PTPRD, PTPRS, PTPRT, QKI, RAB35, RAC1, RAD21, RAD50, RAD51, RAD51B, RAD51C, RAD51D, RAD52, RAD54L, RAF1, RANBP2, RARA, RASA1, RB1, RBM10, RECQL4, REL, RET, RFWD2, RHEB, RHOA, RICTOR, RIT1, RNF43, ROS1, RPS6KA4, RPS6KB1, RPS6KB2, RPTOR, RUNX1, RUNX1T1, RYBP, SDHA, SDHAF2, SDHB, SDHC, SDHD, SETBP1, SETD2, SF3B1, SH2B3, SH2D1A, SHQ1, SLIT2, SLX4, SMAD2, SMAD3, SMAD4, SMARCA4, SMARCB1, SMARCD1, SMC1A, SMC3, SMO, SNCAIP, SOCS1, SOX10, SOX17, SOX2, SOX9, SPEN, SPOP, SPTA1, SRC, SRSF2, STAG1, STAG2, STAT3, STAT4, STAT5A, STAT5B, STK11, STK40, SUFU, SUZ12, SYK, TAF1, TBX3, TCEB1, TCF3, TCF7L2, TERC, TERT, TET1, TET2, TFE3, TFRC, TGFBR1, TGFBR2, TMEM127, TMPRSS2, TNFAIP3, TNFRSF14, TOP1, TOP2A, TP53, TP63, TRAF2, TRAF7, TSC1, TSC2, TSHR, U2AF1, VEGFA, VHL, VTCN1, WISP3, WT1, XIAP, XPO1, XRCC2, YAP1, YES1, ZBTB2, ZBTB7A, ZFHX3, ZNF217, ZNF703, ZRSR2.

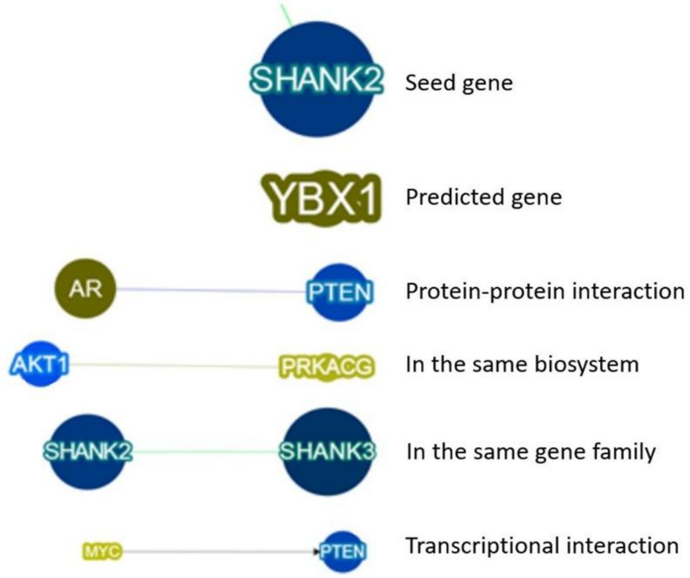

Figure S1. Phenolyzer network visualization legend.

Table S2. Univariate and multivariate analysis of clinical and molecular prognostic factors.

**Co-variate**	**Dicothomization**	**Median survivals**	**No. of events/patients**	**P at univariate**	**HR**	**95% CI**	***P* at multivariate**
Age	<70y vs ≥70y	47 vs 87	38/89 vs 17/38	0.9671	1.01	0·56-1·82	0.8352
Gender	M vs F	42 vs 54	38/82 vs 17/45	0.4258	1.88	0.77-2.01	0.2853
Metastatic involvement	1 site vs >1	32 vs 82	20/36 vs 35/91	0.0017	0.33	0.17-0.66	0.0640
Response to first-line CT	DC vs not DC	23 vs 69	37/101 vs 18/26	0.0023	0.33	0.16-0.67	0.0046

Metastatic status	OM vs PM disease	29 vs NR	16/50 vs 39/77	<0.0001	0.19	0.10-0.35	<0.0001
APC	Wt vs Mut	28 vs 87	24/40 vs 31/87	0.0002	0.25	0.13-0.50	0.0001
RAS	Wt vs Mut	87 vs 39	27/69 vs 28/58	0.0729	1.44	0.80-2.58	0.2191
TP53	Wt vs Mut	32 vs 69	23/43 vs 32/84	0.0624	1.52	0.79-2.92	0.2027
TMB	<10 vs ≥10 mut/mb	45 vs 80	44/92 vs 11/35	0.0554	1.43	0.92-3.04	0.0958

CI: Confidence Interval; DC: Disease Control; F: Female; HR: Hazard Ratio; L: Left; M: Male; mut: KRAS mutations; NR: Not Reached; R: Right.

Table S3. TOP 20 mutations (mutational burden) in polymetastatic disease.

**Gene**	**Total mutations**	**Patients with mutation**	**Prevalence**
ETV1	387	77	0.9746835443
ALK	160	75	0.9493670886
PPARG	143	77	0.9746835443
ROS1	135	52	0.6582278481
ETV6	110	65	0.8227848101
EGFR	107	56	0.7088607595
BRCA1	105	38	0.4810126582
TP53	99	64	0.8101265823
APC	98	57	0.7215189873
PAX3	90	47	0.5949367089
MDC1	76	29	0.3670886076
PREX2	75	65	0.8227848101
SPTA1	74	53	0.6708860759
NCOR1	72	35	0.4430379747
MST1	69	61	0.7721518987

ERCC1	69	60	0.7594936709
CCND3	66	56	0.7088607595
NTRK2	62	43	0.5443037975
BARD1	59	51	0.6455696203
MYB	55	38	0.4810126582

Table S4. TOP 20 mutations (mutational burden) in oligo-metastatic disease.

Gene	Total mutations	Patients with mutation	Prevalence
ETV1	350	50	1.0
ALK	121	48	0.96
PPARG	91	43	0.86
TP53	77	44	0.88
ROS1	76	26	0.52
BRCA1	75	24	0.48
APC	72	44	0.88
ETV6	72	41	0.82
EGFR	66	37	0.74
PAX3	65	27	0.54
MDC1	60	20	0.4
SPTA1	54	33	0.66
PREX2	52	44	0.88
NTRK2	52	26	0.52
PAX8	49	26	0.52

LRP1B	49	25	0.5
IDH2	45	7	0.14
CCND3	42	35	0.7
FAT1	42	24	0.48
BARD1	40	32	0.64
